#  Corpos dissidentes que se reinventam 

**DOI:** 10.1590/0102-311XPT012024

**Published:** 2024-04-22

**Authors:** Romeu Gomes

**Affiliations:** 1 Instituto Nacional de Saúde da Mulher, da Criança e do Adolescente Fernandes Figueira, Fundação Oswaldo Cruz, Rio de Janeiro, Brasil.; 2 Hospital Sírio-Libanês, São Paulo, Brasil.

Pedro Paulo Gomes Pereira já publicou inúmeros artigos e livros que, em geral, articulam referenciais socioantropológicos a questões da saúde coletiva. A corporeidade é uma das categorias analíticas que tematizam a sua produção. No livro *A Invenção do Impossível: Gênero e as Poéticas de Abertura*^1^, baseado no seu trabalho de progressão para Professor Titular de Antropologia da Universidade Federal de São Paulo, ele reflete sobre sua trajetória intelectual ao longo de sua carreira como pesquisador. De forma clara e objetiva, consegue enfrentar o desafio de, simultaneamente, introduzir o leitor na reflexão acerca de uma rica experiência em pesquisa e situá-lo nos marcos teórico-conceituais que lhe serviram de ancoragem.

Logo na introdução, o autor usa da estratégia de causar incômodo ao afirmar que “*gênero não está para cultura como sexo está para a natureza*” (p. 21). Com isso, procura desestabilizar posições conformadas sobre o assunto, estimulando os leitores a questionarem qualquer concepção que considere sexo e gênero como substâncias permanentes. Além disso, explicita sua trajetória metodológica, que se afigura numa antropologia interpelada, partindo de teorias, rompendo com elas, recuperando-as e dando-lhes novas direções. Adverte, com base em Lévi-Strauss, que é por meio da experiência do campo de pesquisa que o conhecimento terá o sentido que lhe falta. Nessa trajetória de aprender a aprender, ele coloca o foco sobre corpos dissidentes que se esboçam da arte de se reinventar, ligados a itinerários terapêuticos e cuidado em saúde. Ao longo do livro, essa arte corporal vai adquirindo maior visibilidade por meio da paleta de sentidos que o autor lhe atribui.

O capítulo que se segue é uma problematização sobre a categoria gênero. Nele, o autor se contrapõe às lógicas da colonialidade, apresentando experiências políticas, econômicas e de produção do conhecimento. Por meio de questionamentos, o autor busca colocar em foco viagens de corpos e teorias que almejam se transformar e se redefinir. Nessas viagens, resgata a história da travesti Cilene e também promove uma viagem de conceitos. Ambas se descolam do Norte global e se abrem para corpos reconstruídos e conceitos libertadores.

A terceira parte é uma iniciação ao debate da poética de corpos, materializada no corpo da travesti. Esse corpo - que tanto se encorpora (se modifica) como se incorpora (abre-se à alteridade) - vai tomando sentido e sendo aprendido por meio de um rico diálogo entre medicina e misticismo. Como observa o autor, “*os corpos das travestis têm fronteiras permeáveis, como movimentos acontecendo na carne, e outros, além da pele, numa interação intensa entre pessoas, objetos, saberes, entes*” (p. 75). Se não fosse pela violência que atravessa esses corpos, a sua poética nos causaria prazer em adentrá-la.

Na parte que se segue, a escrita é tecida pelas dimensões espacial, corporal e gestual para entender as poéticas das subjetividades dissidentes. Para isso, o autor recorre a pesquisas que se interconectam em sua produção realizada ao longo das duas últimas décadas. Nessa parte, ele não cai na armadilha de discorrer isoladamente sobre espaço, corpo e gesto. Ao contrário, procura demarcar as inextricáveis relações entre essas dimensões, remetendo-se à multiplicidade de corpos que resistem, reinventam-se e expõem algo sobre gênero, rompendo com “*a aceitação melancólica do desaparecimento*” (p. 97).

Na quinta parte, o autor focaliza aberturas poéticas estancadas pela violência, crimes cometidos contra travestis são narrados, com base em dados secundários e recortes de suas pesquisas etnográficas. Em nota final do capítulo, observa que as violências acompanham todo o percurso das travestis, “*desde quando começam a transformar os corpos, nas escolas, nas famílias, nos serviços de saúde, mesmo depois de mortas*” (p. 122).

Ao final do livro, Pereira retoma a reflexão sobre gênero e sexualidade. Nessa retomada, empreende uma crítica acerca da teoria queer, advertindo que ela “*não surgiria no passado nem no porvir, mas a descobriríamos em forças fora do tempo*” (p. 125), apontando para uma abertura para o que está fora ou para a experiência do fora, em movimentos que apontam para a invenção do impossível. Em síntese, o autor encerra com a abertura para compreender os corpos insurgentes, em específico, e para a reinvenção ou ressignificação da performática de gênero, em geral.

O encerramento, na parte final do livro, é coerente com uma das referências teóricas adotadas pelo autor. Trata-se da questão do devir, central para a filosofia de Deleuze. Segundo esse filósofo, o devir não é imitação, nem assimilação de um modelo. Também não é um ponto a que se chega ou para o qual se parte. É, antes de tudo, próprio do desejo, implicando na consideração de que desejar é passar por devires ^2^.

Se olharmos para os corpos das travestis, tratados no livro, e parafrasearmos Deleuze ^3^, podemos ver esses corpos como minoria. Mas não se trata necessariamente de uma minoria em termos de número, mas no sentido do que está fora do modelo de gênero/sexo, sendo entendido como um devir, um processo, que se desvia de formas estabelecidas e busca para si seus próprios modelos.

Os itinerários das travestis abordados por Pereira muito se assemelham aos retalhos de história ou histórias retalhadas que compõem o pujante livro de Villada ^4^, sobre vidas de travestis. Nesses retalhos, espelha-se o drama de pessoas que, por imposições sociais, não conseguem ser o que desejam. São pessoas, como sublinha a autora, que nem tiveram a oportunidade de se esconderem, porque já nasceram expulsas do armário, escravas de sua aparência.

As histórias de Pereira e as histórias de Villada nos fazem questionar as fronteiras entre ficção e realidade. Os corpos dissidentes estão no ponto em que uma começa e a outra termina, ou onde elas se superpõem. O conhecimento deles permanece ausente ou invisibilizado em diferentes espaços da área da saúde. No âmbito da formação profissional, por vezes, esses corpos sequer são questionados. Já no campo das políticas, apesar de haver brechas para que sejam inseridos, parecem não existir na tradução das ações programáticas. E quando teimam em existir, na busca de cuidados em saúde, muitas vezes, forçam-se a entrar em outro modelo de corpo para conseguirem algum atendimento.

Independentemente do campo de especialização na área da saúde, o profissional que se dispuser a ler esta obra certamente será afetado pela discussão contundente empreendida pelo autor. Os corpos das travestis, em geral, possivelmente continuarão dissidentes, mas não serão mais desconsiderados ou ignorados pelos leitores deste livro.
